# High-efficiency and durable V–Ti–Nb ternary catalyst prepared by a wet-solid mechanochemical method for sustainably producing acrylic acid *via* acetic acid–formaldehyde condensation†

**DOI:** 10.1039/d2ra06960a

**Published:** 2023-01-06

**Authors:** Jun Liu, Youjun Yan, Meng Lian, Jimei Song, Yongqi Yang, Guofu Huang, Miao Wang, Xinzhen Feng, Weijie Ji

**Affiliations:** a Shandong Engineering Laboratory for Clean Utilization of Chemical Resources, Weifang University of Science and Technology Weifang 262700 China; b Key Laboratory of Mesoscopic Chemistry, MOE, School of Chemistry and Chemical Engineering, Nanjing University Nanjing 210023 P. R. China fxz@nju.edu.cn

## Abstract

Based on the precise phase control V species adjustment of vanadium phosphorus oxides (VPOs), a series of metal oxides (Nb_2_O_5_, MoO_3_, WO_3_, and Bi_2_O_3_) were selected as modification agents to further enhance the catalytic activity and retain the excellent durability of VPO–TiO_2_-based catalysts for the new procedure of producing acrylic acid *via* acetic acid–formaldehyde condensation. At an elevated liquid hourly space velocity (LHSV), the (AA + MA) selectivity reached 92.3% with a (MA + AA) formation rate of 63.8 μmol^−1^ g_cat_^−1^ min^−1^ over the Nb-decorated catalyst (catalyst VTi–Nb), and it maintained good durability for up to 100 h. The detailed characterization results of XRD, Raman, XPS, NH_3_-TPD, CO_2_-TPD, and H_2_-TPR, demonstrated that the addition of Nb_2_O_5_ could observably enhance the catalytic efficiency of the VPO–TiO_2_ catalyst. It not only improved the catalyst durability by enhancing prereduction of the V^5+^ species, but also enhanced the active site density to improve the catalytic activity.

## Abstract 2

High-efficiency and durable V–Ti–Nb ternary catalyst prepared by a wet-solid mechanochemical method to enhance efficient condensation of HAc/HCHO to AA/MA.

## Graphical abstract

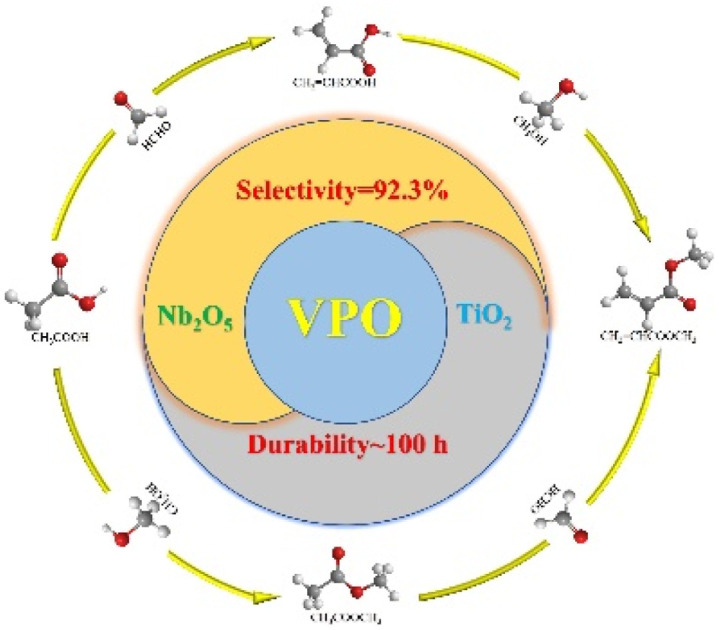

## 1 Introduction

As large-scale chemicals, acrylic acid (AA) and methyl acrylate (MA) are widely used in the production of hydrogels, plastics, adhesives, high-quality paints and varnishes.^1–7^ Presently, the mainstream production process of AA (MA) is a two-step oxidation of propylene.^8–11^ However, petroleum-based propylene feedstock has been considerably affected by the world oil market. This drawback cannot be ignored, even though the superiority of the mainstream production process in terms of economic efficiency and simplicity of device design has been demonstrated. As a result, the motivation to develop a fungible and sustainable procedure for AA and MA preparation is becoming stronger.

For the past few years, a new procedure to prepare AA (MA) by aldol condensation between acetic acid (HAc) and formaldehyde (HCHO) has been widely studied by academia and industry,^12–20^ because raw materials such as HAc and HCHO can be obtained from sustainable biomass or low-cost natural gas, and coal. In fact, the aldol condensation reaction between HAc and HCHO conforms to an acid–base catalytic mechanism,^21,22^ and multifarious types of acid–base catalysts such as alkali metals supported on silica-based materials,^23^ ionic liquids,^24^ acidic zeolites,^14^ and vanadium phosphorus oxide (VPO)^25^ have been developed and applied to the target condensation reaction.

Vanadium phosphorus oxide (VPO), a typical multifunctional catalyst, was originally used as a catalyst for the oxidation of *n*-butane to maleic anhydride, and then used as a catalyst for other reactions such as the dehydration of glycerol and selective oxidation of 5-hydroxymethylfurfural.^5,26–31^ In the 1980s, it was first used to catalyse the aldol condensation reaction between HAc and HCHO as an acid–base bifunctional catalyst by Ai. Thereafter, Ji *et al.*^3,22^ researched the influence of the VPO phase composition on the target condensation reaction and developed a green, low-cost, and extremely simplified catalyst fabrication method. Yin and coworkers^32^ revealed that zeolite supports such as SBA-15 and HZSM-5 have a notable influence on VPO catalyst specific areas, surface acid–base properties and catalytic performance for target reactions. Zhao *et al.*^33^ researched the influence of different oxide supports on VPO catalysts by loading VPO on Nb_2_O_5_, TiO_2_, SiO_2_, ZrO_2_, Sb_2_O_3_, and η/γ-Al_2_O_3_ by the impregnation–deposition method, and discovered that the VPO texture and surface acid–base properties could be dramatically optimized, when η/γ-Al_2_O_3_ was used as a support.

Proverbially, metal cation doping was an effective method for enhancing catalyst activity and durability.^1^ Some metal cation-doped VPO catalysts have been applied for condensation reactions. Li *et al.*^34^ tried to add metal cations into a VPO catalyst by an equivalent-volumetric impregnation method and observed that a 2% Zr-doped VPO catalyst showed the highest activity for the target reaction. Zhu *et al.*^10,13^ provided a variety of metal cation-doped VPO catalysts *via* an organic solvent method and found that the VPO catalyst surface acidity and catalytic performance for the target reaction could be modified by metallic additives such as Mo, W, Cr, La, Ce, and Nb.

In our previous study,^16,25^ the wet-solid mechanochemical method proved its usefulness in the preparation of VPO catalysts. It is not only possible to effectively enhance the catalytic performance by modulating the VPO phase constitution, but also stabilize the V^4+^/V^5+^ redox couple and enhance the catalytic persistence by adding quantized TiO_2_ as a decorating agent. However, previous studies have demonstrated that the medium strong acid site density was directly proportional to surface V^5+^ content and determined the HAc conversion.^16,22,25^ Thus, although the addition of TiO_2_ decorating agent made the V^4+^/V^5+^ redox couple more stable and enhanced the catalyst durability, the reduction of medium acid site density caused by pre-reduction of V^5+^ also leads to a loss of HAc conversion. Based on this identification, a ternary catalyst was designed in the present work, wet-solid mechanochemical methods were still used to introduced additional metal auxiliaries into the VPO–TiO_2_ catalysts to replenish the medium strong acid sites lost through pre-reduction. A series of metal oxides (Nb_2_O_5_, MoO_3_, WO_3_, and Bi_2_O_3_) were selected as modification agents and added into the typical wet-solid mechanochemical process to further enhance the catalytic activity and retain the excellent durability of VPO–TiO_2_-based catalysts for the new route to produce acrylic. Notably, the VPO particles were further broken into 35–40 nm sizes, and the metallic elements were attached to the VPO particles. The diverse exposure tendency for metallic elements (Bi, Mo, Nb, and W) on the catalyst surface led to various impacts on the catalyst textural properties as well as the catalytic performance. Furthermore, characterization by XRD, XPS, Raman spectroscopy, H_2_-TPR, NH_3_-TPD, CO_2_-TPD, and catalytic evaluation indicated that the addition of Nb_2_O_5_ not only improved the catalyst durability by enhancing prereduction of the V^5+^ species, but also enhanced the active site density as well as the catalytic activity.

## 2 Experimental

### 2.1 Materials

Benzyl alcohol (99.9%), phosphoric acid (H_3_PO_4_, 85%), vanadium pentoxide (V_2_O_5_), PEG-6000, cyclohexane, titanium dioxide (TiO_2_, hydrophilic, 100 nm), niobium pentoxide (Nb_2_O_5_, 99.9%), molybdenum trioxide (MoO_3_, 99.95%), tungsten trioxide (WO_3_, 99.9%), bismuth trioxide (Bi_2_O_3_, 99.9%), and acetic acid (99.5%) were purchased in AR. The commercial HCHO solution (37%) contained 10% methanol as a stabilizer.

### 2.2 Catalyst preparation

The preparation of raw γ-VOPO_4_ and δ-VOPO_4_ phases was obtained from literature.^16^ 4.8 g of V_2_O_5_ was added into 72 mL of benzyl alcohol, then refluxed at 140 °C. 5 h later, 2.1 g of PEG-6000 was introduced. After 1 h, 85% phosphoric acid was slowly added to achieve the *P*/*V* atomic ratio of 1.05/1.0, followed by refluxing for 6 h. The solids were filtered out and washed with acetone, then dried in 100 °C air for 24 h to obtain catalyst precursor.

The prepared method of δ-VOPO_4_ phase, 5 g of catalyst precursor was loaded into the reactor, then calcined at 400 °C for 15 h under a flowing O_2_ atmosphere (40 mL min^−1^).

The prepared method of γ-VOPO_4_ phase, 5 g of catalyst precursor was loaded into the reactor, then calcined at 680 °C for 12 h under a flowing atmosphere (75%-O_2_/N_2_, 60 mL min^−1^).

The metal oxide (Nb_2_O_5_, MoO_3_, WO_3_, and Bi_2_O_3_) decorated VPOs with the content being 10%, were obtained through wet-solid mechanochemical method (the wet-solid mechanochemical method was enforced by a mechanical ball-milling process). The mass ratio for γ-VOPO_4_, δ-VOPO_4_, and TiO_2_ (w/w/w = 1 : 3 : 14) as well as the technical parameter of preparation were in accordance with previous literature.^25^ Stoichiometric γ-VOPO_4_, δ-VOPO_4_, TiO_2_, and metal oxide (Nb_2_O_5_, MoO_3_, WO_3_, and Bi_2_O_3_) were added into a 50 mL agate jar, then 50 tiny agate balls were added and 25 mL cyclohexane was served as milling medium. Finally the mixture was ball-milled for 12 h.

### 2.3 Characterization methods

Various techniques including scanning electron microscopy (SEM), X-ray powder diffraction (XRD), XPS, Raman, NH_3_-TPD, CO_2_-TPD, and H_2_-TPR were used to characterize the physical–chemical properties of the catalysts. The detailed information is described in the ESI.†

### 2.4 Catalyst evaluation

The catalytic performance was tested in a fixed bed microreactor at atmospheric pressure. Before the evaluation, all powdery catalysts were pressed, crushed, and sieved to 20–40 mesh. 3 g of catalyst was charged into the reactor, and the space above was filled with quartz sand to preheat the in-coming liquid. The reaction temperature and carrier flow rate was 360 °C and 50 mL min^−1^ (3.0 vol% O_2_ in N_2_), respectively. The liquid feed was a HAc–HCHO mixture (2.5/1, *n*/*n*), with a LHSV of 1.33 mL h^−1^ g_cat._^−1^. The products in 2.5 h were collected in a cold trap, then analyzed by a gas chromatograph with flame ion detector (FID) and HP-FFAP capillary column. Valeric acid and iso-butyl alcohol were used as internal standards for component quantification. In addition, the off-gas was analyzed by TCD and TDX-01 packed column, the unreacted HCHO content was analyzed by the iodometry method. Besides, the methyl acetate (MAc) was not regarded as a harmful by-product. In fact, it can continue to react with HCHO to produce AA/MA, thus there is no negative impact on a recycling manufacture process. For this reason, the molar quantity of generated MAc was treated as unreacted HAc when calculating the HAc conversion and (MA + AA) selectivity (HAc-based).

Formation rate of AA + MA (FR_AA+MA_) is defined by eqn (1):

1 FR_AA+MA_ = *n*_(AA+MA)_/(*m*_VPO_ × *t*)

where *n*_AA+MA_ is the sum of molar quantity of (AA + MA), *m*_VPO_ is the mass quantity of VPO component in the sample, and *t* is the reaction time (150 min).

Selectivity of (AA + MA) (*S*_AA+MA_) based on HAc is defined by eqn (2):

2 *S*_AA+MA_ = *n*_(AA+MA)equ._/(*n*_(HAc)0_−*n*_(HAc)measured_−*n*_(MAc)measured_) × 100%

where *n*_(AA+MA)equ._ is the molar quantity of HAc equivalent to (AA + MA), *n*_(HAc)0_ is the molar quantity of HAc fed into the reactor, *n*_(HAc)measured_ is the molar quantity of unreacted HAc, and *n*_(MAc)measured_ is the molar quantity of generated MAc.

Conversion of HAc (*X*_HAc_) is defined by eqn (3):

3 *X*_HAc_ = (*n*_(HAc)0_−*n*_(HAc)measured_−*n*_(MAc)measured_)/*n*_(HAc)0_ × 100%

where *n*_(HAc)0_ is the molar quantity of HAc fed into the reactor, *n*_(HAc)measured_ is the molar quantity of unreacted HAc, and *n*_(MAc)measured_ is the molar quantity of generated MAc.

The carbon balance is calculated by eqn (4):

4 CB = (*N*_acetone_ × *n*_acetone_ + *N*_MAc_ × *n*_MAc_ + *N*_methanol_ × *n*_methanol_ + *N*_MA_ × *n*_MA_ + *N*_HAc_ × *n*_HAc_ + *N*_AA_ × *n*_AA_ + *N*_HCHO_ × *n*_HCHO_ + *N*_CO_ × *n*_CO_ + *N*_CO_2__ × *n*_CO_2__)_measured_/(*N*_HAc_ × *n*_(HAc)0_ + *N*_HCHO_ × *n*_(HCHO)0_ + *N*_methanol_ × *n*_(methanol)0_) × 100%

where *N* is the number of carbon in a specific molecule, *n* is the mole quantity of each component measured by GC and titration.

## 3 Results and discussion

### 3.1 Morphology analysis

**Fig. 1:**
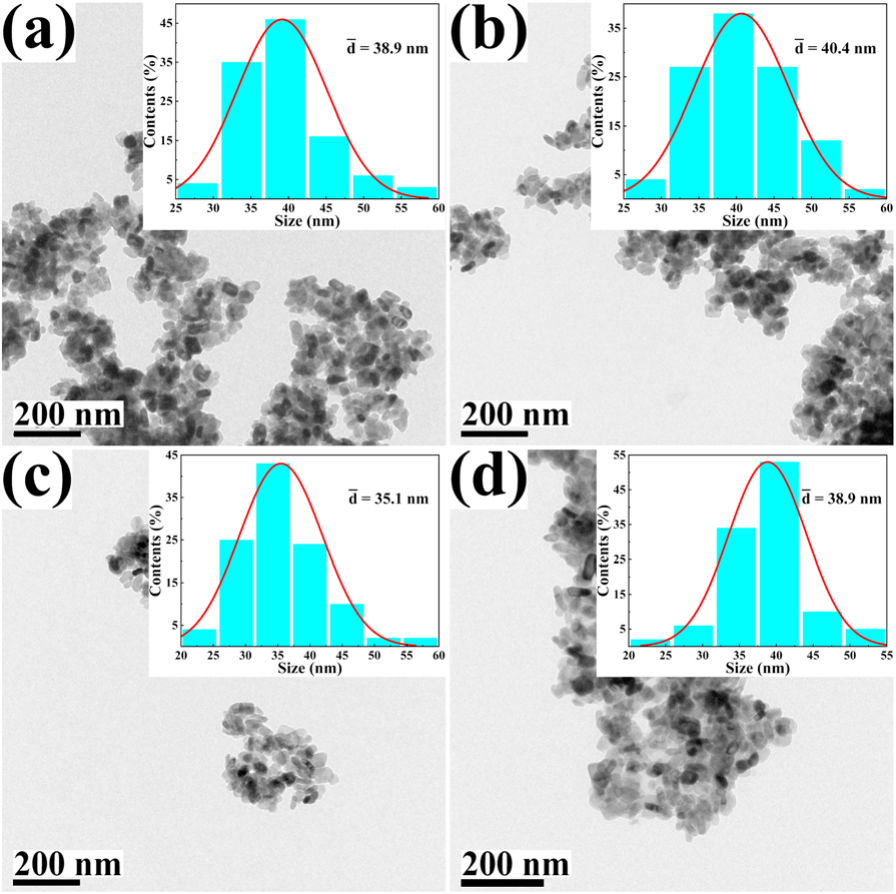
TEM images of metal cation-doped VPO catalysts: (a) VTi–Bi, (b) VTi–Mo, (c) VTi–Nb, and (d) VTi–W.

**Fig. 2:**
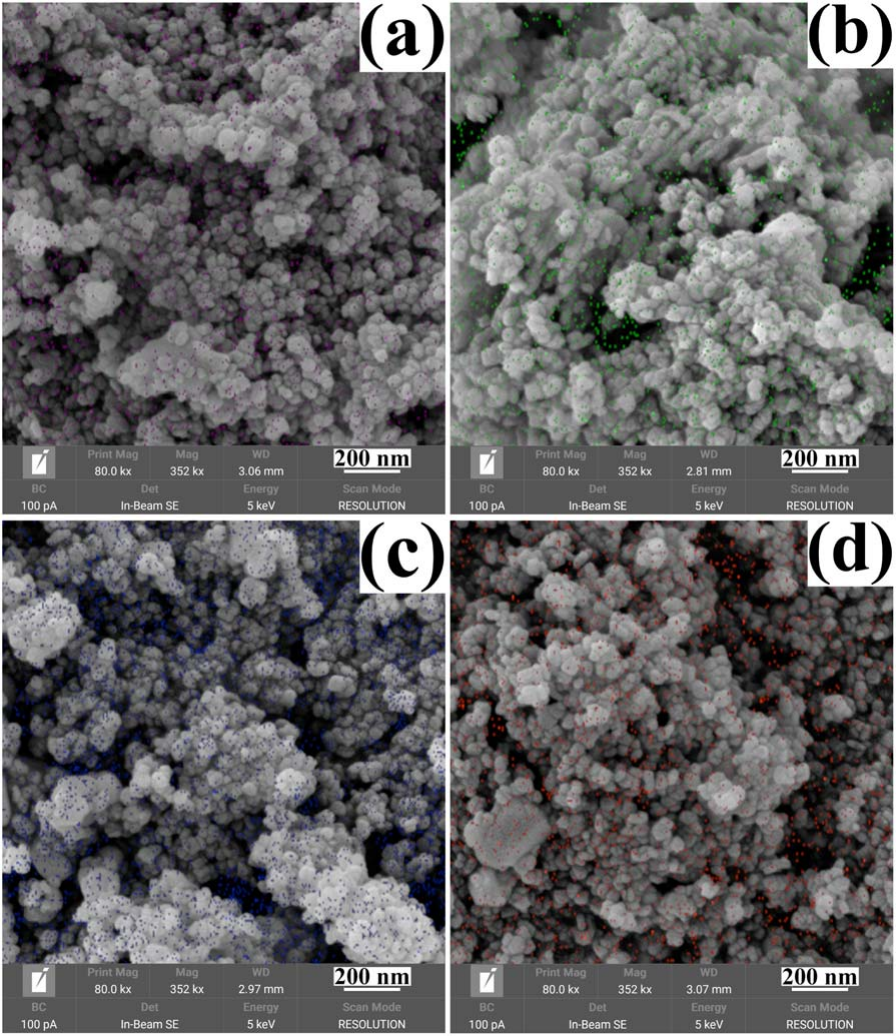
SEM images of metal cation-doped VPO catalysts: (a) VTi–Bi, (b) VTi–Mo, (c) VTi–Nb, and (d) VTi–W. The magenta, green, blue, and orange dots were respectively corresponding to the distribution of Bi, Mo, Nb, and W elements on the catalyst surface measured by EDX mapping.

The morphology and size of the metal cation-decorated VPO catalysts were characterized by TEM and SEM, the results were shown in Fig. 1 and 2. In contrast to previous reports,^16,25^ the average particle size of metal cation-decorated VPO catalysts prepared by wet-solid mechanochemical method was 35–40 nm, although all of the catalysts still showed fine particle morphology. It was evident that the size of the VPO particles was further reduced, based upon the interaction of the wet-solid mechanochemical process, TiO_2_, and the addition of metal cations. This means that the particles would be in closer contact with each other, and the interfacial interaction would be further enhanced. In addition, the distribution of metallic elements (Bi, Mo, Nb, and W) on the catalysts surface was characterized by EDX mapping, and the results were shown in Fig. 2. Obviously, the surface densities of metallic elements including Bi, Mo, Nb, and W were clearly different, even though the doping ratios were identical. Catalyst VTi–Bi had the sparsest distribution, while the catalyst VTi–Nb was significantly denser than the others. This result indicated that the exposed tendency for metallic elements on the catalyst surface was diverse, and even lead to various impacts on the catalyst textural properties.

### 3.2 XRD analysis

**Fig. 3:**
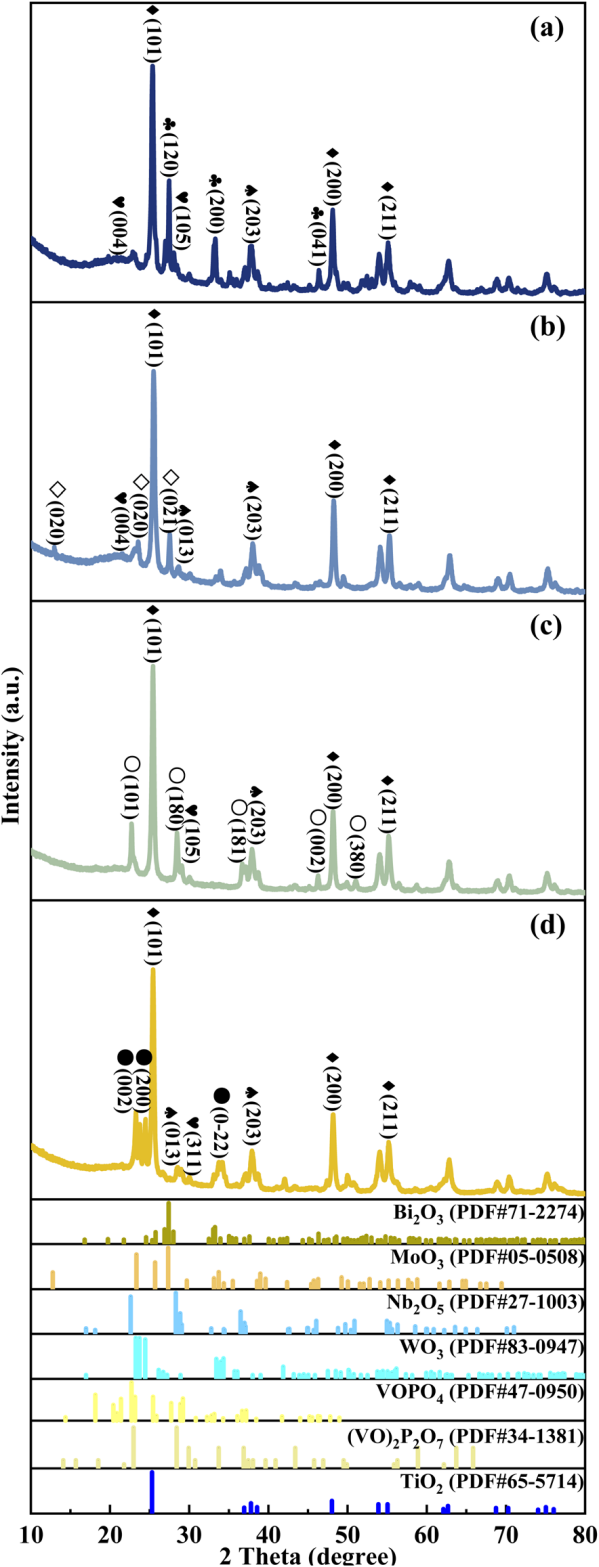
XRD patterns of metal cation-doped VPO catalysts: (a) VTi–Bi, (b) VTi–Mo, (c) VTi–Nb, and (d) VTi–W. 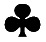 Bi_2_O_3_, ◇MoO_3_, ○Nb_2_O_5_, ●WO_3_, ♦TiO_2_, 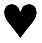 VOPO_4_, 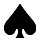 (VO)_2_P2O_7_.

The crystal texture of metal cation-decorated VPO catalysts was analysed by X-ray diffraction. The results and the standard PDF(s) of the related reference crystals were shown in Fig. 3. According to the standard PDF(s), the main diffraction lines for Bi_2_O_3_ (at 2*θ* = 27.6°, 33.4°, and 46.6°), MoO_3_ (at 2*θ* = 12.8°, 23.6°, and 27.5°), Nb_2_O_5_ (at 2*θ* = 22.7°, 28.4°, and 36.6°), WO_3_ (at 2*θ* = 23.3°, 23.7°, and 24.5°), and TiO_2_ (at 2*θ* = 25.3°, 48.0°, 53.9°, and 55.1°) can be clearly seen in Fig. 3. However, the XRD patterns of VPOs were distinctly changed. The main diffraction lines for δ-VOPO_4_ (2*θ* = 19.5°, 22.0°, 24.2°, and 28.5°) were instead by (VO)_2_P_2_O_7_ (2*θ* = 22.8°, 28.3°, 36.7°, and 43.2°). This result indicated that the VPO phases were clearly transformed, while the crystal texture of metallic oxide additives such as Bi_2_O_3_, MoO_3_, Nb_2_O_5_, WO_3_, and TiO_2_ remained unchanged.

### 3.3 Raman analysis

**Fig. 4:**
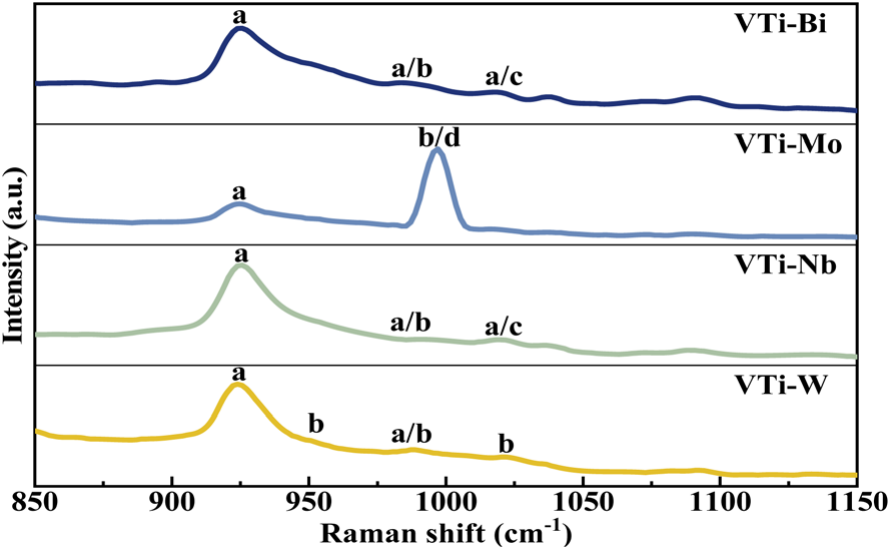
Raman spectra of metal cation-doped VPO catalysts included VTi–Bi, VTi–Mo, VTi–Nb, and VTi–W. (a) (VO)_2_P_2_O_7_, (b) γ-VOPO_4_, (c) δ-VOPO_4_, and (d) MoO_3_.

The VPO phase composition could be effectively analysed *via* Raman spectra, on the basis of our previous studies.^35^ As shown in Fig. 4, the representative bands of (VO)_2_P_2_O_7_ (at 921 cm^−1^) distinctly appeared in every line,^36^ yet the bands for δ-VOPO_4_ (at 1020 cm^−1^) and γ-VOPO_4_ (at 996 and 1022 cm^−1^) were weak and blurry, indicating that the main VPO entity after the mechanical ball-milling process was (VO)_2_P_2_O_7_, rather than γ-VOPO_4_ and δ-VOPO_4_. In accordance with the XRD analysis, the addition of metallic oxides such as Bi_2_O_3_, MoO_3_, Nb_2_O_5_, and WO_3_ played a role in helping the further reduction of the pentavalent VPO entity.

### 3.4 XPS analysis

**Fig. 5:**
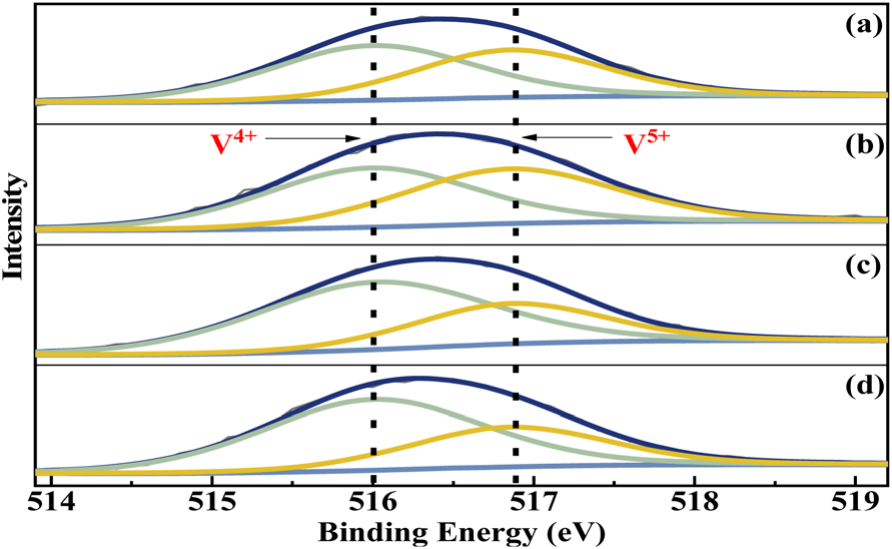
V 2p_3/2_ curve fitting analysis of metal cation-doped VPO catalysts: (a) VTi–Bi, (b) VTi–Mo, (c) VTi–Nb, and (d) VTi–W.

The elemental composition and the vanadium oxidation state at the surface of the metal cation-decorated VPO catalysts were measured by XPS. The O 1s, P 2p, Mo 3d, Bi 4f, W 4f, and Nb 3d spectra were shown in Fig. S1–S3,† respectively. And as shown in Fig. 5, the V 2p spectra of all metal cation-decorated VPO catalysts could be deconvolved into two separate peaks. The peak located at 516.0 eV belonged to V^4+^ 2p_3/2_,^37^ while the peak centred at 516.9 eV belonged to V^5+^ 2p_3/2_.^38^ The results of the deconvolution analysis and surface elemental composition are summarized in Table 1. Compared to previous studies, the V^4+^/V^5+^ ratios of catalysts VTi–Nb (2.02) and VTi–W (1.97) were higher than those of catalysts without additives (1.60),^25^ and the V^4+^/V^5+^ ratios of catalysts VTi–Mo (1.07) and VTi–Bi (1.23) were lower, indicating that the addition of Nb_2_O_5_ and WO_3_ enhanced the reduction reaction between VPO and cyclohexane, while MoO_3_ and Bi_2_O_3_ impaired it. In fact, the relationship between the prereduction of the V^5+^ species and durability of the VPO catalyst has been confirmed.^25^ Therefore, in theory, the addition of Nb_2_O_5_ could further slow the reduction rate of surface V^5+^ in the reaction process and improve the durability of the VPO–TiO_2_ catalyst. In addition, the X/V ratios (X= Nb, Mo, W, Bi) of the metal cation-decorated VPO catalysts were significantly different, and the exposed density of Nb on the catalyst surface was the highest, while the exposed density of Bi was considerably below the others, agreeing with the SEM results. The distinguishing metal cation exposure density could affect the catalyst surface acidity, to varying degrees.

**Table 1:** XPS results derived of metal cation-doped VPO catalysts

Catalysts	*P*/*V* ratio	V^4+^/V^5+^ ratio	X/V ratio (X = Nb, Mo, W, Bi)
VTi–Nb	1.9	2.02	0.42
VTi–Mo	1.9	1.07	0.10
VTi–W	1.8	1.97	0.10
VTi–Bi	1.8	1.23	5.86 × 10^−3^

### 3.5 NH_3_- and CO_2_-TPD analysis

**Fig. 6:**
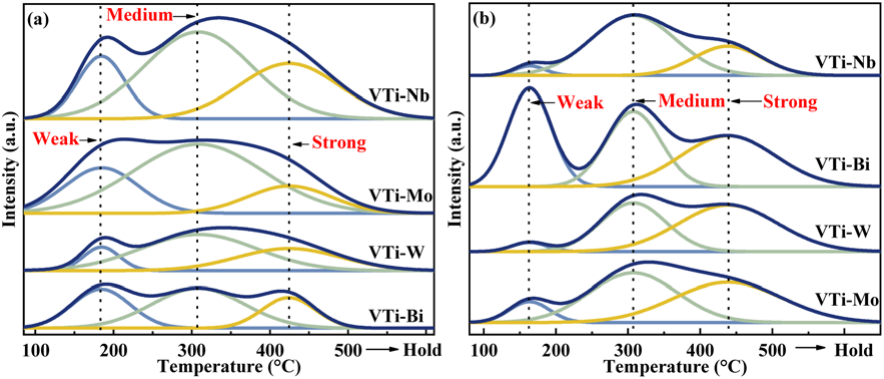
NH_3_- and CO_2_-TPD profiles of metal cation-doped VPO catalysts: (a) NH_3_-TPD, (b) CO_2_-TPD.

The surface acidity/basicity was studied by NH_3_- and CO_2_-TPD, because the target aldol condensation reaction is a typical acid-base catalysed reaction. The NH_3_- and CO_2_-TPD curves for all of the metal cation-decorated VPO catalysts are shown in Fig. 6. The NH_3_-TPD curves could be deconvolved into three peaks, which apartly corresponded to the weak (183 °C), medium (307 °C), and strong (424 °C) acid sites,^13^ and the three peaks for CO_2_-TPD curves corresponded to the weak (161 °C), medium (307 °C), and strong (439 °C) base sites.^39^ The acid and base sites densities were calculated by calibrating the corresponding NH_3_ and CO_2_ desorption peak area, and the results are recorded in Table S1 (ESI†). Undoubtedly, the medium acid site density of the catalyst VTi–Nb was slightly higher than that of the catalyst VTi–Mo and considerably greater than that of the catalysts VTi–W and VTi–Bi. However, in theory, the order of the medium acid site density on the VPO surface should be similar to the order of the V^5+^/V^4+^ ratios (Table 1), because the literature^13,22,25^ show that the surface V^5+^ specimen is the main factor determing the medium acid site density. The situation was caused by the differential metal cation exposure density. The highest Nb element exposure density on the catalyst VTi–Nb led to the highest medium acid site density, although the V^5+^/V^4+^ ratio was significantly lower than others. However, the exposure of Nb elements to the catalyst surface also led to abundant strong acid sites and enhanced side reactions. This explained why the catalyst VTi–Nb exhibited a slightly lower (MA + AA) selectivity (HAc-based) than the catalyst VTi–Mo, even though it has the highest medium base site density. It had been reported that the (MA + AA) selectivity (HAc-based) for ordinary VPOs catalysts was extremely consistent with the *P*/*V* ratio and medium base site density.^3,16,25,40^

### 3.6 H_2_-TPR analysis

**Fig. 7:**
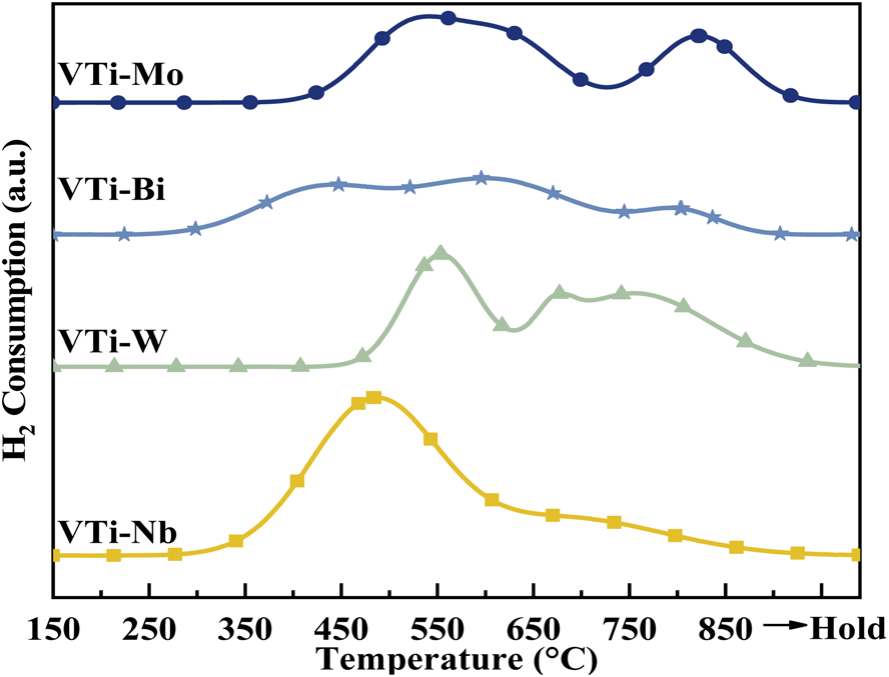
H_2_-TPR profiles of metal cation-doped VPO catalysts.

According to literature,^16,25,33^ the catalytic performance, especially catalyst durability, could be affected by the activity of lattice oxygen on the catalyst surface. Therefore, the surface lattice oxygen activity of the metal cation-decorated VPO catalysts was researched by H_2_-TPR, and the results are recorded in Fig. 7 and Table S2 (ESI†). Evidently, the locations of the reduction peaks were affected by the addition of metal cations. Especially for the Nb and Bi decorated catalysts, the initial reduction peaks were brought down to 500 °C, while all of the primordial reduction peaks for the undecorated catalyst should be higher than 530 °C.^25^ Furthermore, as shown in Table S2 (ESI†), the total H_2_ consumption for catalyst VTi–Nb was significantly higher than that for the other catalysts, which was caused by the enrichment and reduction of Nb on the catalyst surface. The lower reduction peak and highest H_2_ consumption implied that the surface lattice oxygen of catalyst VTi–Nb was more active than that of other catalysts, which also indicated that the addition of Nb could significantly enhance the catalyst durability.

### 3.7 Catalytic performance

**Fig. 8:**
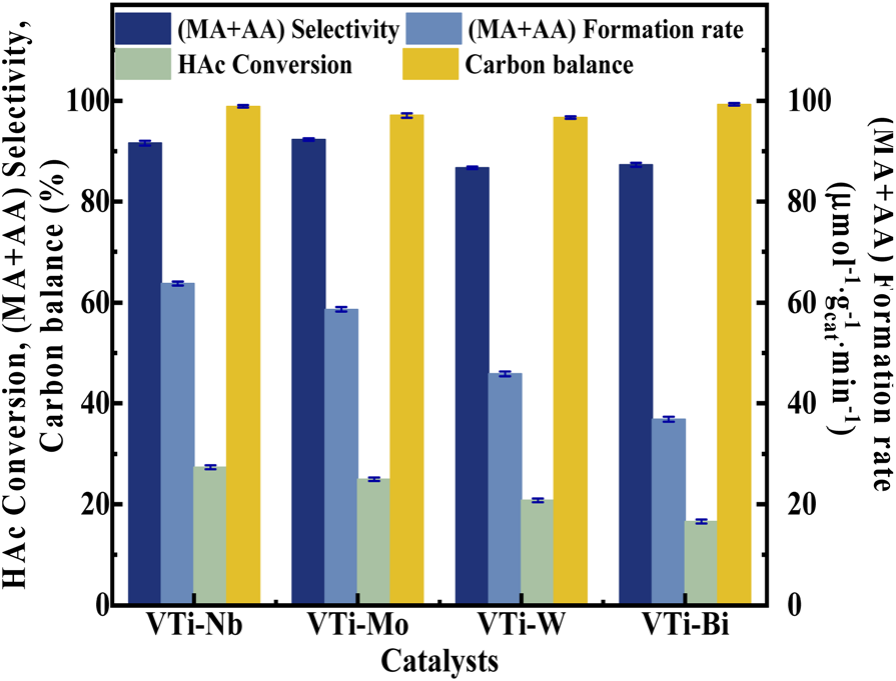
Catalyst performance of metal cation-doped VPO catalysts. The reaction temperature and carrier flow rate was 360 °C and 50 mL min^−1^ (3.0 vol% O_2_ in N_2_), respectively. The liquid feed was a HAc–FA mixture (2.5/1, *n*/*n*), with a LHSV of 1.33 mL h^−1^ g_cat._^−1^.

Various metal cation-decorated VPO catalysts were evaluated at atmospheric pressure for the aldol condensation reaction between HAc and HCHO, and the reaction parameters are detailed in the SI. As shown in Fig. 8, the catalyst VTi–Nb showed the highest HAc conversion (27.4%), followed by VTi–Mo, VTi–W, and VTi–Bi, in order with the medium acid site density. However, unlike previous reports,^25,40^ the (MA + AA) selectivity was out of order with the medium base site density order. The highest (MA + AA) selectivity (92.3%) occurred in catalyst VTi–Mo, rather than catalyst VTi–Nb. The reason is the abundant strong acid sites on the catalyst VTi–Nb surface caused by the exposed Nb-enhanced side reactions reducing the (MA + AA) selectivity. However, in terms of the (MA + AA) formation rate, the catalyst VTi–Nb still maintained the best catalytic activity, which was significantly higher than that of the other metal cation-decorated VPO catalysts. It is also notable that the LHSV of the raw solution was 1.33 mL h^−1^ g_cat._^−1^ in the catalyst evaluation process, almost four times that of the LHSV in a previous study,^25^ whereas the HAc conversion was still approximately 27%, similar to the catalyst VPO–TiO_2_ without metal cation-decoration. Furthermore, the (MA + AA) selectivity reached 92.3%, far surpassing previous studies.^16,22,25,40^ Thus, the HAc conversion did not decrease due to the increased LHSV under the catalyst VTi–Nb, indicating that the addition of Nb_2_O_5_ enhanced the active site density as well as the catalytic efficiency.

**Fig. 9:**
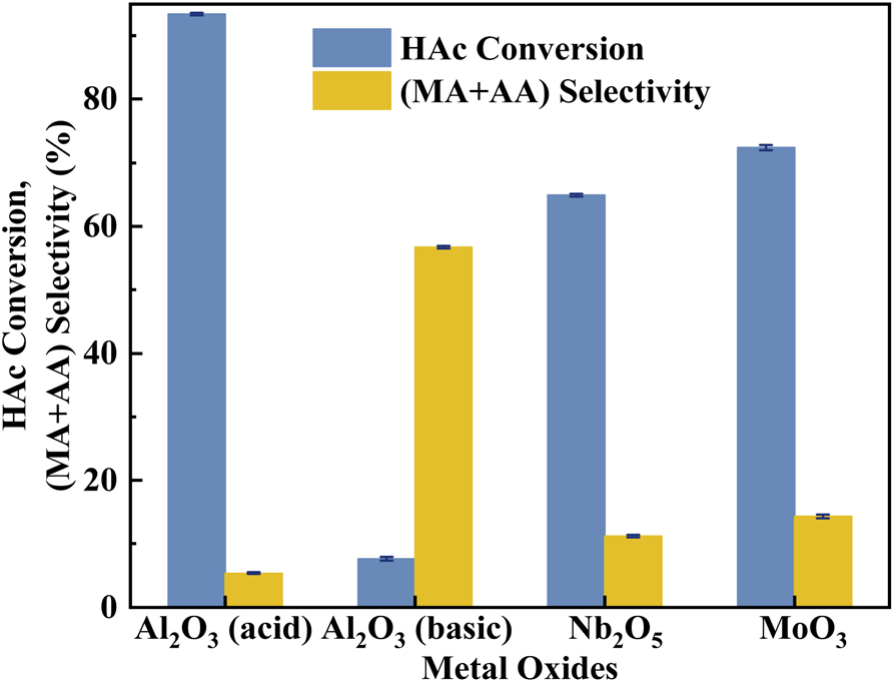
Catalyst performance of metal coxides. The reaction temperature and carrier flow rate was 360 °C and 50 mL min^−1^ (3.0 vol% O_2_ in N_2_), respectively. The liquid feed was a HAc–FA mixture (2.5/1, *n*/*n*), with a LHSV of 1.33 mL h^−1^ g_cat._^−1^.

According to the literature,^41^ the activation of α-H of acetic acid was directly related to the surface acidity/basicity. Therefore, a set of controlled experiments were conducted to evidence the role of acidity/basicity in delivering maximum selectivity and conversion. Pure Nb_2_O_5_, MoO_3_ as well as the typical solid acid/base catalysts, acid alumina and basic alumina were evaluated under the same conditions for the aldol condensation reaction between HAc and HCHO. As shown in Fig. 9, the HAc conversion on Al_2_O_3_ (acid) was the highest (93.4%), then followed by MoO_3_ (72.4%)and Nb_2_O_5_ (64.9%), in order with the surface acid site density (Fig. S4 and Table S3†). Similarly, the (MA + AA) selectivity on Al_2_O_3_ (basic) was the highest (56.7%), then followed by MoO_3_ (14.3%)and Nb_2_O_5_ (11.2%), in order with the surface base site density (Fig. S5 and Table S3†). Besides, the HAc conversion on Al_2_O_3_ (basic) and (MA + AA) selectivity on Al_2_O_3_ (acid) were minimal, indicated that extremely acid or basic catalysts were not beneficial to the target reaction. Obviously, the surface acidity/basicity were the key factor affecting the HAc conversion and (MA + AA) selectivity respectively.

For the metal cation-decorated VPO catalysts in current study, a structure–function relationship between catalytic performance and catalyst structural properties could be established. Firstly, as the TEM and SEM results showed, the initial particles were smashed even smaller,^25^ compared with the VPO–TiO_2_ catalyst without additional metal auxiliaries. It means the particles would be in closer contact with each other, and the interfacial interaction would be further enhanced. And the promotion effect of the interfacial interaction on the activity of VPO catalysts has been confirmed in previous reports.^16^ Secondly, systematically analyzed the results of XRD, Raman, XPS, NH_3_-TPD, CO_2_-TPD, and H_2_-TPR demonstrated that change in types of metal oxides in the series of ternary catalysts led to the transformations in VPO phase composition, surface V species and acid–base properties, and then influenced the catalytic behavior. The addition of Nb_2_O_5_ further enhanced the prereduction reaction between V^5+^ and cyclohexane as well as the interfacial interaction between VOPO_4_ and (VO)_2_P_2_O_7_. In theory, the pre-reduction of V^5+^ would lead to a loss of surface medium acid site density as well as a decrease of HAc conversion, even though it has a positive effect on the durability of VPO catalyst for the target reaction.^25^ However, the acidity of Nb_2_O_5_ itself and the intense tendency of exposure on the catalyst surface make-up for the loss by obtaining extra medium acid sites on the surface. Therefore, the addition of Nb_2_O_5_ could observably enhance the catalytic efficiency of VPO–TiO_2_ catalyst, including both the catalyst durability and catalytic activity.

### 3.8 Catalyst durability

**Fig. 10:**
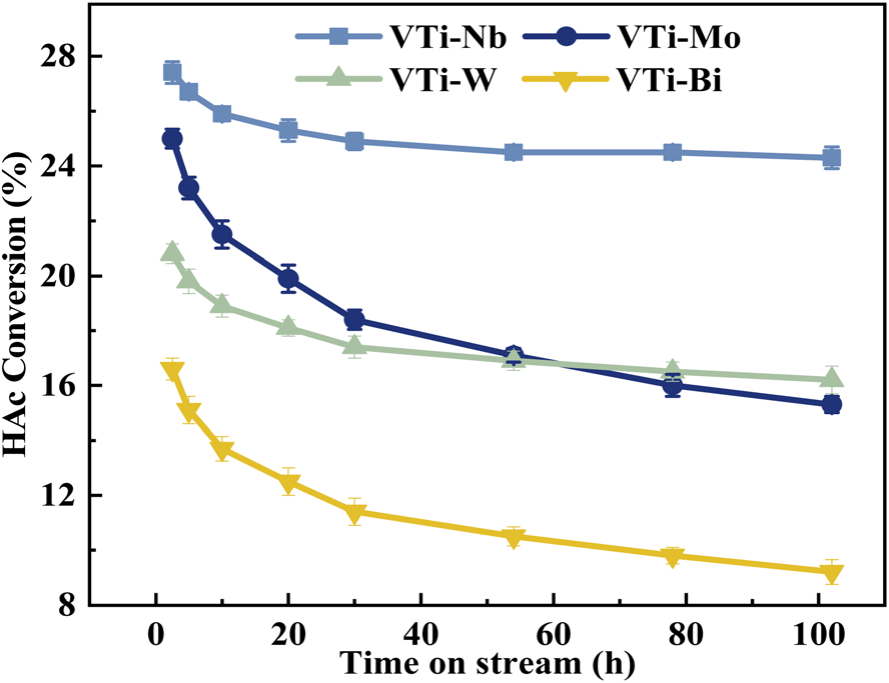
Durability of HAc conversion test over the metal cation-doped VPO catalysts in TOS of 100 h.

**Fig. 11:**
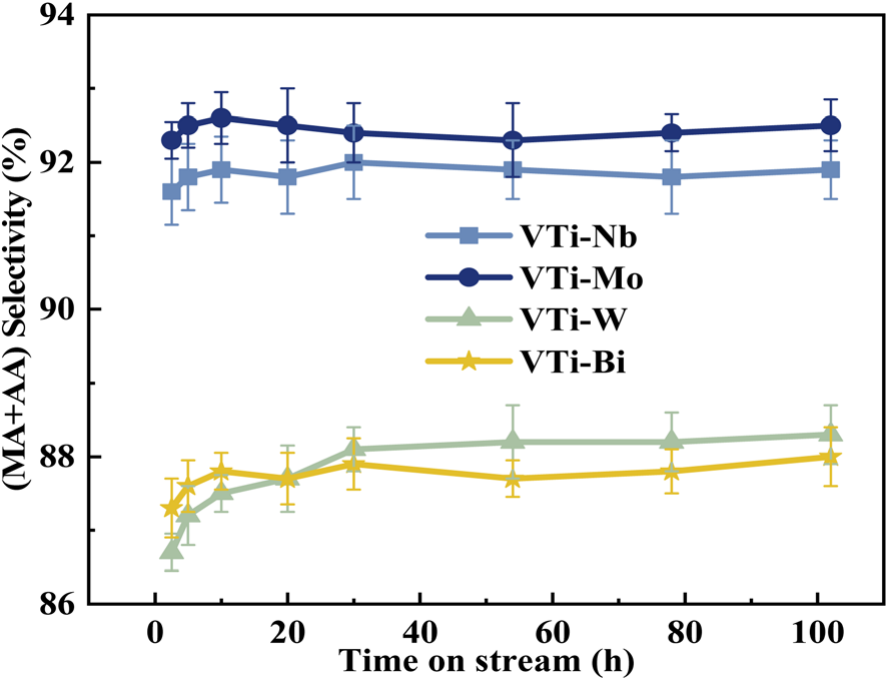
Durability of (MA + AA) selectivity test over the metal cation-doped VPO catalysts in TOS of 100 h.

The durability of various metal cation-decorated VPO catalysts was examined at the same reaction parameters for approximately 100 h. As shown in Fig. 10, 11, S6 and S7,† the HAc conversion and (MA + AA) formation rate for all the metal cation-decorated VPO catalysts decreased rapidly in the first 30 h and then fell extremely slowly in the following 100 h. Meanwhile, the (MA + AA) selectivity reached the highest in the first 30 h and then practically maintained in the following 100 h. This indicated that the HAc conversion could be the primary cause of the drop in the (MA + AA) formation rate, instead of the (MA + AA) selectivity. The spent catalysts were analyzed by means of NH_3_-TPD and XPS. As shown in Fig. S8† and Table 2, in a time-on-stream of 100 h, the surface acidity of all metal cation-decorated VPO catalysts decreased, but the medium acid site density of catalyst VTi–Nb was still the highest, then followed by VTi–W, VTi–Mo, and VTi–Bi, which was consistent with the HAc conversion. And the XPS results also indicated that the catalyst VTi–Nb was the best for durability. As shown in Fig. S9† and Table 3, with the progress of the reaction, the V^5+^ on the catalysts surface were further reduced, among which the reduction degree of catalyst VTi–Mo was the highest, the V^4+^/V^5+^ ratio increased from 1.07 to 3.76, then followed by VTi–W and VTi–Bi. While the reduction degree of catalyst VTi–Nb was the lowest, in a time-on-stream of 100 h, the V^4+^/V^5+^ ratio on catalyst surface only increased by 0.17 (from 2.02 to 2.19), indicating that the addition of Nb_2_O_5_ could observably enhance the stability of V^4+^/V^5+^ redox couple as well as the durability of catalyst. In addition, a significant feature was observed that the decrease rates of catalyst VTi–Nb was slower than others, due to the prereduction of the V^5+^ species and the excellent surface lattice oxygen activities. It is also evident in the carbon balance data, catalyst VTi–Nb remained at approximately 99% in the TOS of 100 h, while other catalysts always remained below 98%. According to early studies,^16,25,40^ the primary reason for deactivation was the reduction of surface V^5+^ and inactivation of lattice oxygen. Thus, the prereduction of the V^5+^ species, durable lattice oxygen and abundant active sites make VTi–Nb a significantly better catalyst than the others.

**Table 2:** The surface acidity of spent catalysts

Catalysts	Acid site distribution (μmol NH_3_ g_cat._^−1^)			Total acidity (μmol NH_3_ g_cat._^−1^)
	Weak	Medium	Strong	
VTi–Nb	31.6	119.7	57.3	208.6
VTi–W	18.1	91.9	6.3	116.3
VTi–Mo	7.0	56.1	20.4	83.5
VTi–Bi	22.1	36.7	14.7	73.5

**Table 3:** XPS results derived of the spent catalysts

Catalysts	*P*/*V* Ratio	V^4+^/V^5+^ Ratio	X/V Ratio (X = Nb, Mo, W, Bi)
VTi–Bi	1.8	2.57	6.12 × 10^−3^
VTi–Mo	1.9	3.76	0.09
VTi–Nb	1.9	2.19	0.41
VTi–W	1.8	2.89	0.10

## 4 Conclusions

In this study, a series of metal oxides (Nb_2_O_5_, MoO_3_, WO_3_, and Bi_2_O_3_) were selected as modification agents to further enhance the catalytic activity and retain the excellent durability of VPO–TiO_2_-based catalysts for the new route to produce acrylic acid *via* acetic acid–formaldehyde condensation. Compared with the onefold VPO–TiO_2_ catalyst, the particles were further broken into 35–40 nm sizes, and the metallic elements were attached to the VPO particles. Interestingly, the distributed densities were obviously different, depending on the different metallic elements such as Bi, Mo, Nb, and W. The diverse exposure tendency for metallic elements on the catalyst surface led to various impacts on the catalyst textural properties as well as the catalytic performance. The detailed characterization results of XRD, XPS, Raman, NH_3_-TPD, CO_2_-TPD, and H_2_-TPR, demonstrated that the addition of Nb_2_O_5_ could clearly enhance the catalytic efficiency of the VPO–TiO_2_ catalyst. It not only improved the catalyst durability by enhancing prereduction of the V^5+^ species, but also enhanced the active site density as well as the catalytic activity.

## Author Contributions

Jun Liu and Xinzhen Feng designed this research; Meng Lian, Guofu Huang, Jimei Song, Yongqi Yang, Youjun Yan, Miao Wang conducted research; Jun Liu and Guofu Huang analyzed data; Jun Liu and Youjun Yan wrote the paper; Weijie Ji modified the paper; Jun Liu, Xinzhen Feng, and Weijie Ji edited the whole manuscript.

## Conflicts of interest

The authors declare no competing interests.

## Supplementary Material

RA-013-D2RA06960A-s001
