# Supplementary material for: High-efficiency and durable V–Ti–Nb ternary catalyst prepared by a wet-solid mechanochemical method for sustainably producing acrylic acid via acetic acid–formaldehyde condensation
Source: RSC Adv. 2023 Jan 6;13(3):1530–8. doi: 10.1039/d2ra06960a (PMC9816953; doi:10.1039/d2ra06960a)
Supplement: RA-013-D2RA06960A-s001 [file RA-013-D2RA06960A-s001.pdf]

Electronic Supplementary Material (ESI) for RSC Advances.
This journal is © The Royal Society of Chemistry 2022

Supplementary

High-efficiency and DurableV-Ti-Nb Ternary Catalyst Prepared by Wet-solid Mechanochemical Method for Sustainably Producing Acrylic Acid via Acetic Acid–Formaldehyde Condensation

Jun Liu,[a,b] Youjun Yan,[a] Meng Lian,[a] Jimei Song,[a] Yongqi Yang,[a] Guofu Huang,[a] Miao Wang,[a] Xinzhen Feng[*b] and Weijie Ji[b]

a. Shandong Engineering Laboratory for Clean Utilization of Chemical Resources, Weifang University of Science and Technology, Weifang 262700, China.

b. Key Laboratory of Mesoscopic Chemistry, MOE, School of Chemistry and Chemical Engineering, Nanjing University, Nanjing 210023, P. R. China.

Catalyst preparation details

Benzyl alcohol was employed as the preparation media. V2O5 was first refluxed in benzyl alcohol at 140 °C for 5 h, after that certain amount of PEG 6000 was introduced. One hour later, phosphoric acid (85%) was added drop wise to reach a P/V atomic ratio being 1.05/1.0. The suspension was refluxed for another 6 h, and then the solid was filtered out, and washed with acetone. It was further dried at 100 °C in air for 24 h to obtain catalyst precursor.

The precursor was activated at 400 °C for 15 h under a flowing O_2 atmosphere (40 mL/min). The δ-$VOPO_4$ phase were obtained.

The precursor was activated at 680 °C for 12 h under a flowing atmosphere (75%-O_2/N_2, 60 mL/min). The γ-$VOPO_4$ phase were obtained.

Wet-solid mechanochemical method details. The wet-solid mechanochemical method was enforced by a mechanical ball-milling process. In the mechanical ball-milling process, stoichiometric γ-$VOPO_4$, δ-$VOPO_4$, TiO_2, and metal oxide (Nb_2O_5, MoO_3, WO_3, and Bi_2O_3) were added into a 50 mL agate jar, then 50 little agate balls were added and 25 mL cyclohexane was served as milling medium, finally the mixture was ball-milled for 12 h.

Characterization details

Raman. The Raman spectra were recorded at RT on an HORIBA LabRAM HR Evolution Raman spectrometer (laser source: 532 nm).

XRD. X-ray powder diffraction (XRD) patterns were recorded on a Philips X'Pert MPD Pro X-ray diffractometer with graphite monochromatized Cu Ka radiation (k = 0.1541 nm).

XPS. The binding energy (BE) was calibrated against the C1s signal (284.6 eV) of contaminant carbon. Elemental surface composition was estimated on the basis of peak areas normalized using Wagner factors. Relative surface concentration of V element with different oxidation state can be estimated through deconvolution analysis of the corresponding XPS peak. For the same batch of sample measured under identical conditions as well as the same parameters adopted for deconvolution analysis, the

V^{4+}/V^{5+} ratio of different samples is obtainable for comparison.

H_2-TPR. Hydrogen temperature-programmed reduction (H_2-TPR) was performed from room temperature (RT) to 850 °C at a rate of 10 °C/min in a flow of 5% H_2/Ar (v/v, flow rate = 40 mL/min) and isothermally held at 850 °C until reduction was complete.

NH_3 and CO_2-TPD. Catalyst of 50 mg was first heated in an Ar flow (40 mL/min) to 200 °C and kept at this temperature for 1 h. Then the sample was cooled to 100 °C in the Ar flow. After that, NH_3 or CO_2 adsorption was performed at 100 °C for 1 h. Finally, NH_3 and CO_2-TPD was carried out in an Ar flow (40 mL/min) with the sample being heated to 500 °C at a rate of 10 °C/min. The amount of desorbed NH_3 (in μmol/g) was determined by a titration, in which a HCl solution (0.01 mol/L) was used to absorb the released NH_3. A NaOH solution (0.01 mol/L) was used as the titrant. And the calcium oxalate was used as a Standard to calculated the amount of desorbed CO_2 (in μmol/g).

Catalyst evaluation details

All the catalyst powders were pressed, crushed, and sieved to 20-40 mesh for activity evaluation. Two reactors were used for catalyst evaluation, one has an ID of 10 mm without a thermocouple jacket, and the other has an ID of 12 mm with a thermocouple jacket whose outside diameter is 3 mm. The reaction data derived from the two reactors were proved to be reproducible. Catalyst of 3 g was charged into the reactor, and the space above the catalyst bed was filled with quartz chips to preheat the in-coming liquid. Before feedstock introduction, the sample was heated up in a flow of N_2 (30 mL/min) to a desired temperature (360 °C) at a rate of 10 °C/min and kept at this temperature for 2.5 h. When a mixed HAc and FA solution (2.5/1, n/n) was fed into the reactor with a LHSV of 1.33 $mL{\cdot}h^{-1}{\cdot}g$-1 cat (15.25 $mmol{\cdot}h^{-1}{\cdot}g$-1 cat, HAc-based), a mixture of N_2 and air (50 mL/min, 3 vol.% O_2 in N_2) was served as carrier gas. The products were collected in a cold trap. After 2.5-h reaction, the collected liquid sample was analyzed using a gas chromatograph equipped with a flame ion detector (FID) and a HP-FFAP capillary column (0.32 mm × 25 m). Valeric acid and iso-butyl alcohol

were used as internal standards for component quantification. All the catalysts were first evaluated by screening their performances in terms of the (MA + AA) Yield in the collected liquid sample based on FA input. In these circumstances, the off-gas was on-line analyzed by a GC equipped with TCD and TDX-01 packed column. It is worth noting that the formaldehyde component cannot be measured by GC analysis, therefore, the formaldehyde conversion cannot be directly determined by using the GC analysis data. In some cases, the unreacted FA content was analyzed by the iodometry method. Note that HAc is usually fed significantly excessive in amount over FA (in the current study the molar HAc/FA is 2.5:1) to obtain an overall better performance, the by-products such as acetone and CO_x are mainly originated from HAc, thus the data associated with HAc conversion and particularly (AA + MA) selectivity based on the converted HAc is informative and meaningful to evaluate process economy. In addition, MAc was not regarded as a harmful by-product. In fact, it can continue to react with FA to produce AA/MA, thus there is no negative impact on a recycling manufacture process. For this reason, the molar quantity of generated MAc was treated as unreacted HAc when calculating the HAc conversion and (MA+AA) Selectivity (HAc-based).

Formation rate of AA+MA (FR_{AA+MA}) is defined by equation S1:

$$FR_{AA+MA} = n_{(AA+MA)}/(m_{VPO} \times t) \qquad \textbf{(S1)}$$

Where n_{AA+MA} is the sum of molar quantity of (AA+MA), m_{VPO} is the mass quantity of VPO component in the sample, and t is the reaction time (150 min).

Selectivity of (AA+MA) (S_{AA+MA}) based on HAc is defined by equation S2:

$$S_{AA+MA} = n_{(AA+MA)equ}/(n_{(HAc)0}-n_{(HAc)measured}-n_{(MAc)measured}) \times 100\% \qquad \textbf{(S2)}$$

Where $n_{(AA+MA)equ}$ is the molar quantity of HAc equivalent to (AA+MA), $n_{(HAc)0}$ is the molar quantity of HAc fed into the reactor, $n_{(HAc)measured}$ is the molar quantity of unreacted HAc, and $n_{(MAc)measured}$ is the molar quantity of generated MAc.

Conversion of HAc (X_{HAc}) is defined by equation S3:

$$X_{HAc} = (n_{(HAc)0}-n_{(HAc)measured}-n_{(MAc)measured})/n_{(HAc)0} \times 100\% \qquad \textbf{(S3)}$$

Where $n_{(HAc)0}$ is the molar quantity of HAc fed into the reactor, $n_{(HAc)measured}$ is the molar quantity of unreacted HAc, and $n_{(MAc)measured}$ is the molar quantity of generated

MAc.

The carbon balance is calculated by equation S4:

$$CB = (N_{acetone} \times n_{acetone} + N_{methyl\ acetate} \times n_{methyl\ acetate} + N_{methanol} \times n_{methanol} + N_{methyl\ acrylate} \times n_{methyl\ acrylate} + N_{acetic\ acid} \times n_{acetic\ acid} + N_{acrylic\ acid} \times n_{acrylic\ acid} + N_{formaldehyde} \times n_{formaldehyde} + N_{CO} \times n_{CO} + N_{CO2} \times n_{CO2})_{measured} / (N_{acetic\ acid} \times n_{(acetic\ acid)0} + N_{formaldehyde} \times n_{(formaldehyde)0} + N_{methanol} \times n_{(methanol)0}) \times 100\% \quad \textbf{(S4)}$$

Where N is the number of carbon in a specific molecule, n is the mole quantity of each component measured by GC and titration.

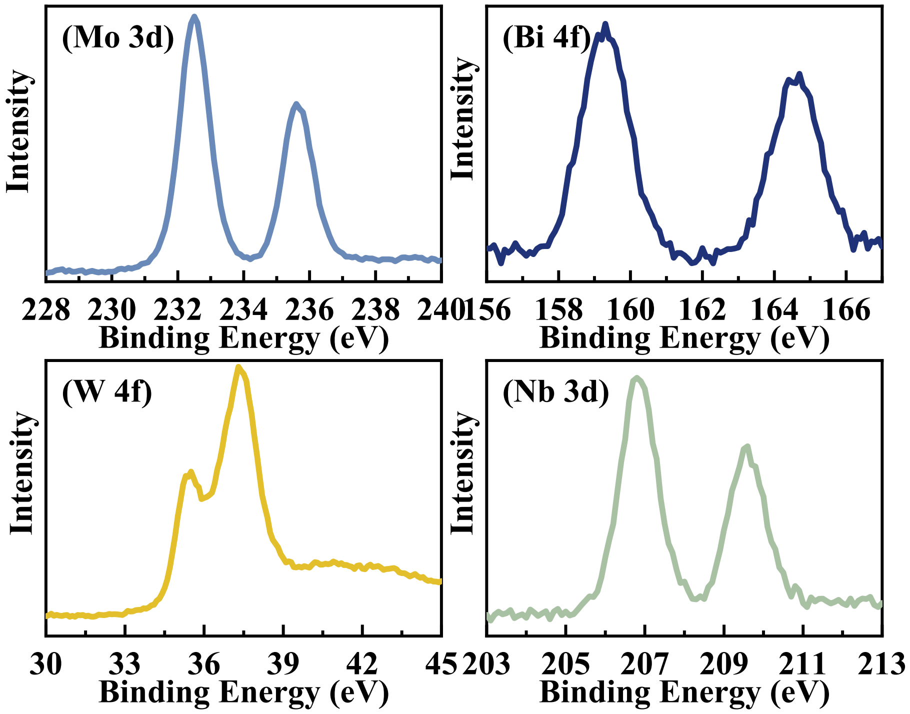

Fig. S1 XPS spectra of Mo 3d, Bi 4f, W 4f, and Nb 3d for the metal cation-doped VPO catalysts, the binding energies of Mo $3d_{5/2}$ and Mo $3d_{3/2}$ were observed at 232.6 and 235.9 eV, the binding energies of Bi $4f_{7/2}$ and Bi $4f_{5/2}$ were observed at 159.3 and 164.6 eV, the binding energies of W $4f_{7/2}$ and W $4f_{5/2}$ were observed at 35.9 and 38.0 eV, the binding energies of Nb $3d_{5/2}$ and Nb $3d_{3/2}$ were observed at 206.9 and 208.5 eV.

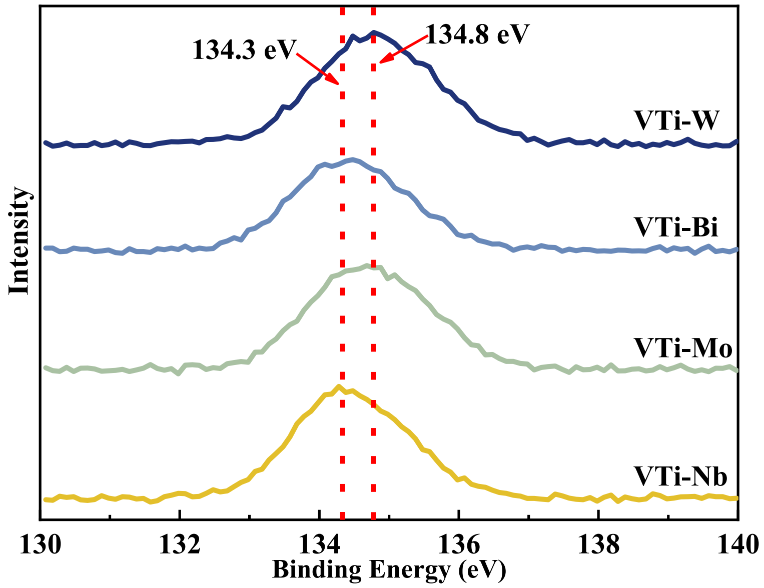

Fig. S2 XPS spectra of P 2p for the metal cation-doped VPO catalysts.

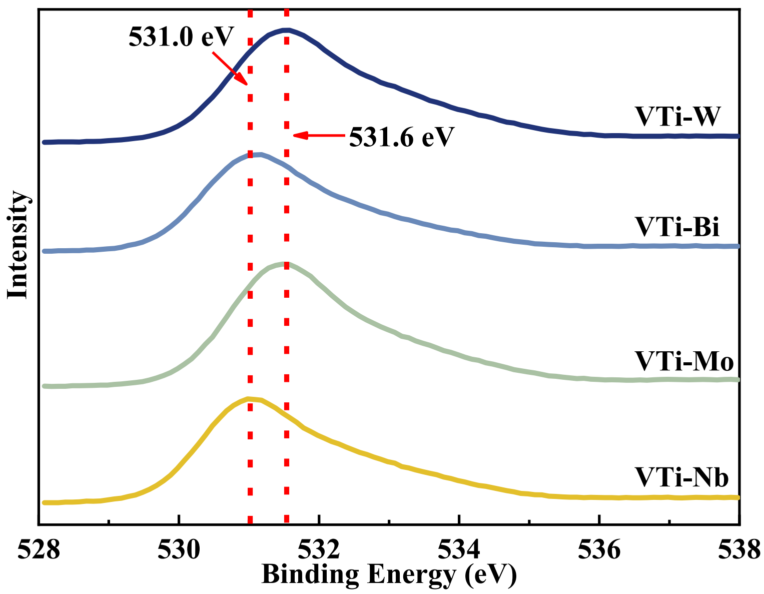

Fig. S3 XPS spectra of O 1s for the metal cation-doped VPO catalysts.

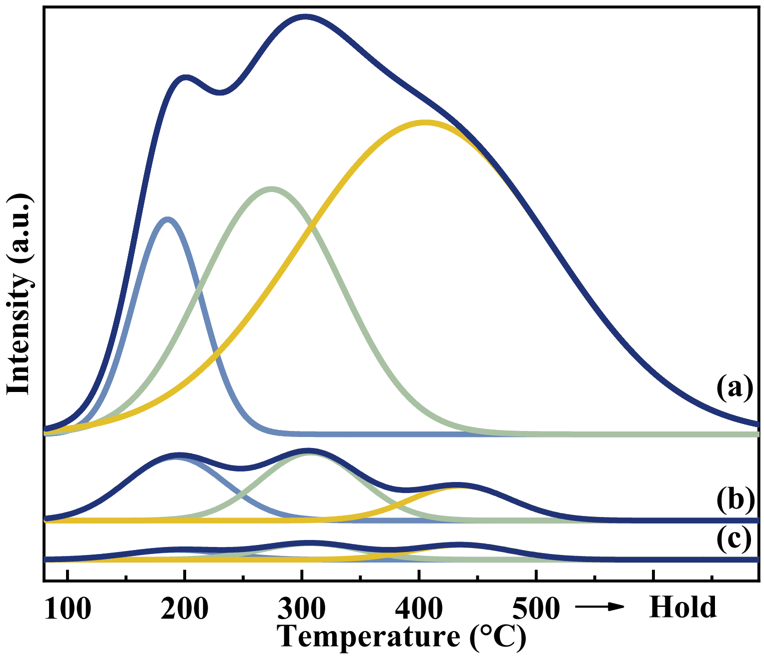

Fig. S4 NH_3 -TPD profiles of metal oxides: (a) Al_2O_3 (acid), (b) MoO_3, (C) Nb_2O_5.

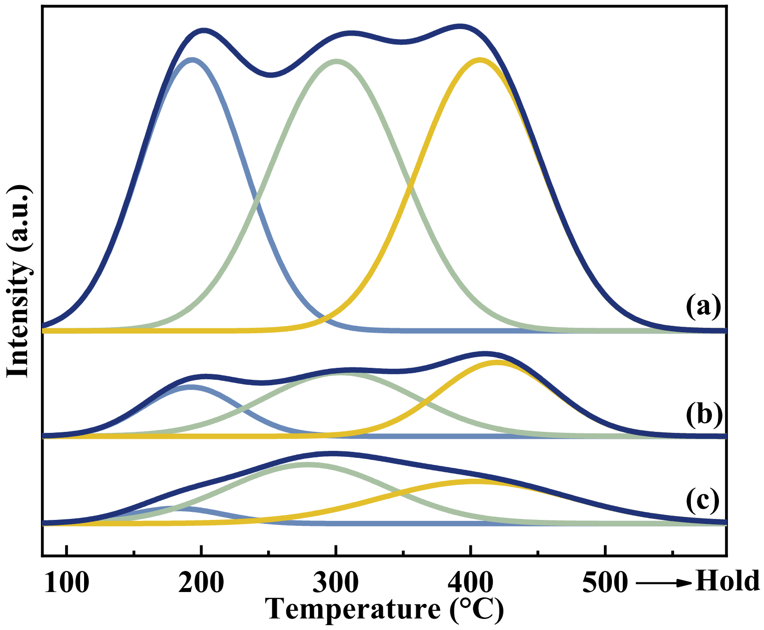

Fig. S5 CO_2 -TPD profiles of metal oxides: (a) Al_2O_3 (basic), (b) MoO_3, (C) Nb_2O_5.

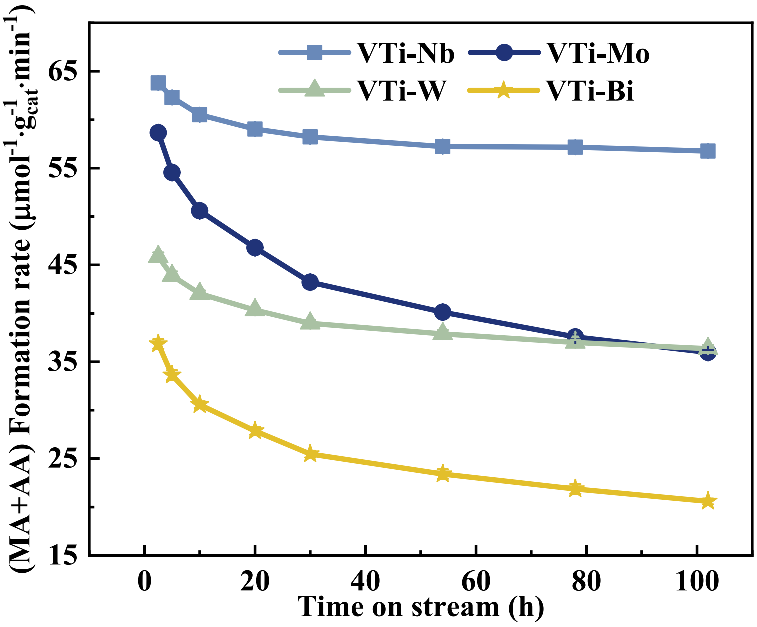

Fig. S6 Durability of (MA+AA) formation rate test over the metal cation-doped VPO catalysts in TOS of 100 h.

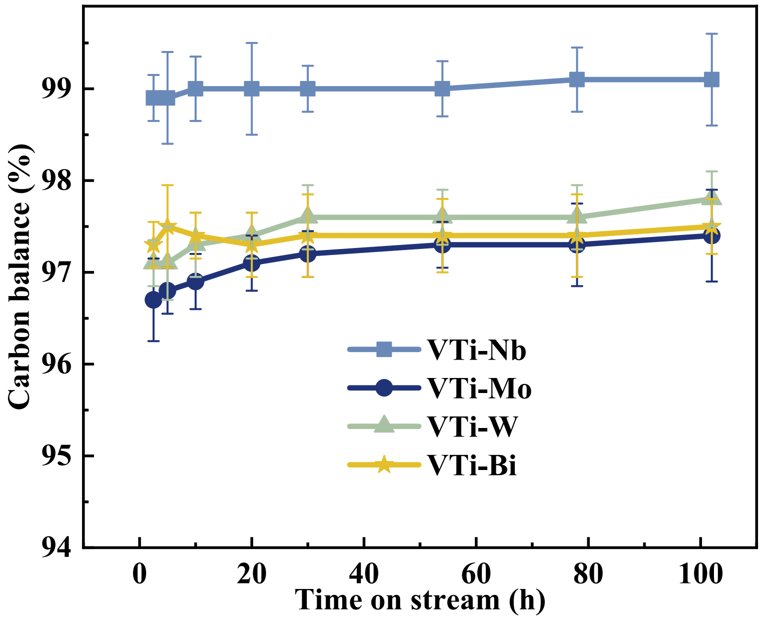

Fig. S7 Durability of carbon balance test over the metal cation-doped VPO catalysts in TOS of 100 h.

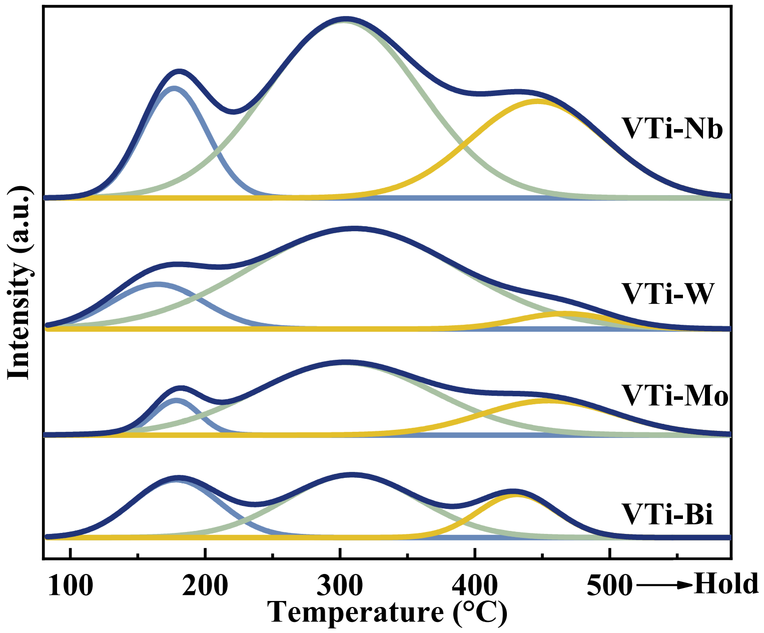

Fig. S8 NH_3 -TPD profiles of the spent catalysts.

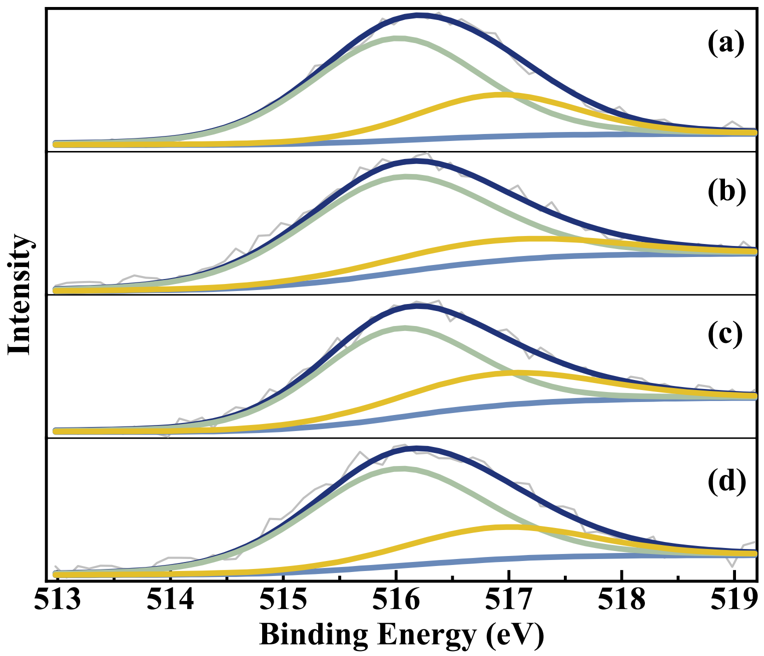

Fig. S9 V 2p3/2 curve fitting analysis of the spent catalysts: (a) VTi-Bi, (b) VTi-Mo, (c) VTi-Nb, and (d) VTi-W.

Table S1 The surface acidity/basicity of metal cation-doped VPO catalysts

**Catalysts**	**Acid site distribution (μmol NH_3/g_{cat})**			**Total acidity (μmol NH_3/g_{cat})**	**Basic site distribution (μmol CO_2/g_{cat})**			**Total Basicity (μmol CO_2/g_{cat})**
	Weak	Medium	Strong		Weak	Medium	Strong	
VTi-Nb	52.3	167.8	98.7	318.8	2.0	33.5	13.2	48.7
VTi-Mo	59.6	164.0	41	264.6	5.7	28.6	28.9	63.2
VTi-W	14.9	75.6	41.1	131.6	2.1	20.9	30.3	53.3
VTi-Bi	40.2	66.3	30.0	136.5	28.0	27.2	31.6	86.8

Table S2 H_2 consumption for V^{5+}/V^{4+} reduction of metal cation-doped VPO catalysts

**Catalysts**	**Total H_2 consumption (mmol H_2/mol V)**
VTi-Mo	15.0
VTi-Bi	12.7
VTi-W	15.7
VTi-Nb	21.2

Table S3 The surface acidity/basicity of metal oxides

**metal oxides**	**Acid site distribution (μmol NH_3/g_{cat})**			**Total acidity (μmol NH_3/g_{cat})**	**Basic site distribution (μmol CO_2/g_{cat})**			**Total Basicity (μmol CO_2/g_{cat})**
	Weak	Medium	Strong		Weak	Medium	Strong	
Al_2O_3 (acid)	218.9	461.8	1141.2	1821.9	\	\	\	\
Al_2O_3 (basic)	\	\	\	\	166.0	204.6	194.4	565.0
MoO_3	88.9	96.4	49.5	234.8	27.1	55.4	49.2	131.7
Nb_2O_5	55.5	91.8	84.0	231.3	8.1	55.1	46.4	109.6
